# Monitoring Adherence and Renal Safety of Nucleos(t)ide Analogs for Patients With Chronic Hepatitis B

**DOI:** 10.14309/ctg.0000000000000962

**Published:** 2025-12-09

**Authors:** Chia-Chen Hsu, Zih-Er Chen, Fan-Hsiu Chao, Chian-Ying Chou, Yuh-Lih Chang, Yueh-Ching Chou, Ming-Chih Hou, Jaw-Ching Wu, Chien-Wei Su

**Affiliations:** 1 Department of Pharmacy, Taipei Veterans General Hospital, Taipei, Taiwan;; 2 Department of Pharmacy, School of Pharmaceutical Sciences, National Yang Ming Chiao Tung University, Taipei, Taiwan;; 3 Department of Medicine, Division of Gastroenterology and Hepatology, Taipei Veterans General Hospital, Taipei, Taiwan;; 4 School of Medicine, College of Medicine, National Yang Ming Chiao Tung University, Taipei, Taiwan;; 5 Institute of Clinical Medicine, College of Medicine, National Yang Ming Chiao Tung University, Taipei, Taiwan;; 6 Department of Medicine, Division of General Medicine, Taipei Veterans General Hospital, Taipei, Taiwan.

**Keywords:** chronic hepatitis B, entecavir, tenofovir, guideline adherence, renal dysfunction

## Abstract

**INTRODUCTION:**

Entecavir (ETV), tenofovir disoproxil fumarate (TDF), and tenofovir alafenamide (TAF) are first-line nucleos(t)ide analogs (NA) for chronic hepatitis B (CHB). Real-world monitoring of patients on these agents and their comparative renal safety remain poorly characterized. We evaluated guideline-adherent monitoring practices and compared renal dysfunction risk across ETV, TDF, and TAF.

**METHODS:**

We retrospectively analyzed patients with CHB who initiated ETV, TDF, or TAF between 2012 and 2022. Levels of serum alanine aminotransferase, total bilirubin, albumin, serum creatinine, hepatitis B virus DNA, and abdominal sonograms were assessed every 6 months during the 36 months of NA treatment. Incidence rates and adjusted hazard ratios (HRs) for renal dysfunction were estimated by Cox regression.

**RESULTS:**

Of the 2,155 enrolled patients, 65.8% received ETV, 23.1% received TDF, and 11.1% underwent TAF. Alanine aminotransferase was monitored in >90% across all groups; other tests (bilirubin, albumin, creatinine, hepatitis B virus DNA, sonogram) were performed in only 20%–80%. After multivariable adjustment, TDF (HR 1.41; 95% confidence interval 0.95–2.08) and TAF (HR 0.91; 95% confidence interval 0.52–2.18) showed no significant difference in renal dysfunction risk vs ETV. Independent predictors of increased renal risk included older age, higher Charlson comorbidity index, fibrosis-4 score, and diuretic use, whereas elevated serum albumin levels were associated with a lower risk.

**DISCUSSION:**

In this real-world cohort, adherence to recommended monitoring for patients with CHB on NAs was suboptimal. ETV, TDF, and TAF demonstrated comparable renal safety profiles over 3 years.

## Graphical abstract

**Figure:**
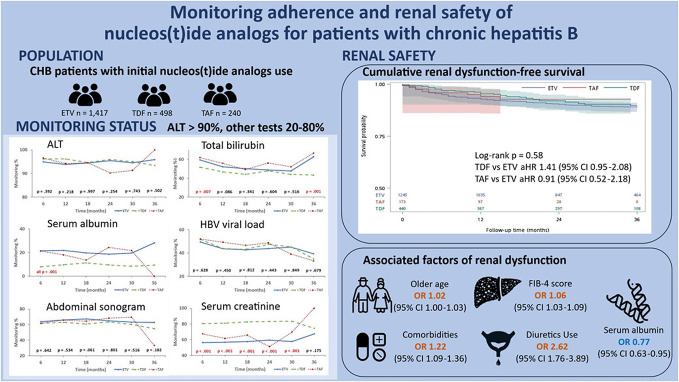

## INTRODUCTION

The worldwide prevalence of chronic hepatitis B virus (HBV) infection was approximately 4.1% in 2019, and an estimated 316 million individuals were affected (1). HBV-related diseases accounted for 555,000 deaths globally in 2019. Antiviral therapy for patients with chronic HBV (CHB) infection effectively reduces the risk of long-term complications such as cirrhosis, liver failure, and hepatocellular carcinoma (HCC) (2–4).

Currently, potent nucleos(t)ide analogs (NAs), including entecavir (ETV), tenofovir disoproxil fumarate (TDF), and tenofovir alafenamide (TAF), are recommended as first-line treatments for CHB (5–7). This recommendation is based on potent antiviral effects and minimal risk of drug resistance during treatment. Although NAs can effectively inhibit HBV replication, they do not eradicate the virus. Discontinuation of NA treatment significantly increases the risk of relapse, particularly in patients who do not achieve hepatitis B surface antigen seroclearance (functional cure) during NA therapy (7,8). Thus, drug usage is often prolonged. The long-term safety of NAs in CHB treatment and patient compliance with the regimen are critical concerns in clinical practice.

Adhering to clinical practice guidelines and maintaining active clinical surveillance are crucial for achieving optimal outcomes in CHB patients. Failure to monitor patients can result in more virological breakthroughs and liver-related events including HCC, cirrhotic complications, and mortality (9). However, studies have revealed that adherence to monitoring guidelines for patients with CHB is suboptimal both during the initial evaluation and over the long term (10,11). For example, Wu et al studied 962 patients with CHB in the United States and demonstrated that 45% did not undergo appropriate HCC screening and 29% did not receive timely laboratory assessment for CHB (10). Nevertheless, information regarding the comparative effectiveness and safety monitoring of the 3 potent NAs, as well as whether clinical guidelines are consistently followed, is limited (12).

A meta-analysis revealed that chronic kidney disease (CKD) was more prevalent among patients with CHB than among the general population (adjusted hazard ratio [HR] 1.40, 95% confidence interval [CI] 1.16–1.69) (13). This highlights the importance of preserving renal function in patients undergoing long-term NA therapy. Despite these imperatives, prior research findings regarding the effects of different NAs on renal function have been inconclusive. TDF has both increased the risk (14–16) and showed no significant difference (17) in the worsening of renal function compared with ETV. TAF, the most recently approved NA, produces less renal toxicity than TDF does. However, although the results of phase 3 clinical trials have indicated an increased risk of TDF (18–20), real-world studies have revealed no significant difference in renal function decline between TDF and TAF (21,22). Moreover, inconsistent outcomes have been observed when TAF was compared with ETV (14,23–25), even in studies conducted in the same country with similar study designs (14,23). To date, no clinical trial has directly compared all 3 agents simultaneously. Recent real-world studies that compared ETV, TDF, and TAF also reported no significant differences in renal function decline during short-term follow-up (26,27). However, these studies were limited by small sample sizes, short observation periods, and the lack of long-term assessment of clinically meaningful renal progression. Thus, the relationship between NAs and renal function in patients with CHB is unclear, which emphasizes the need for further research.

In this study, we assessed the current clinical status of patients undergoing NAs therapy for CHB and compared the risk of renal dysfunction for each of the 3 potent NAs.

## METHODS

### Study design and data source

This retrospective cohort study was conducted at Taipei Veterans General Hospital (TPEVGH), one of the largest medical centers in Taiwan, hosting more than 2.5 million outpatient visits annually and serving approximately 1.1 million patients. Data for this study were obtained from the Big Data Center at TPEVGH. The study adhered to the principles outlined in the Declaration of Helsinki and current ethical guidelines. The study was approved by the Institutional Review Board of TPEVGH (IRB-TPEVGH No.: 2021-09-020BC). The requirement for informed consent was waived by the board because of the retrospective observational nature of the study, and patient data were anonymized before analysis.

### Study population

The study included outpatients aged 20 years or older who had received their initial prescription of ETV, TDF, or TAF for CHB and had continued treatment for more than 84 days between January 1, 2012, and March 31, 2022. The date of the first prescription for NA treatment was designated as the index date. In the initial phase of the analysis (part 1), we excluded patients with concurrent human immunodeficiency virus infection, hepatitis C virus infection, hepatitis D virus infection, HCC diagnosed within 3 months of the index date, a history of organ transplantation, prescription of NAs to prevent HBV reactivation during antitumor or immunosuppressive therapy, and a documented history of previous NA usage. These exclusions were applied because such conditions could independently affect treatment selection and renal outcomes or involve different clinical indications and monitoring protocols compared with CHB therapy.

In the subsequent stage of analysis (Part 2), we additionally excluded patients with kidney-related diseases and those whose kidney-related data were incomplete. Eligible participants were classified into 3 groups according to the specific NA drug prescribed on the index date: ETV, TDF, or TAF. Patients with missing renal data (serum creatinine or estimated glomerular filtration rate (eGFR)) were excluded from the primary analysis because renal dysfunction was the main outcome of interest, and missing key laboratory values could lead to outcome misclassification.

### Ascertainment of outcomes

In Part 1 of this study, we observed monitoring practices regarding various clinical parameters, including levels of serum alanine aminotransferase (ALT), total bilirubin, albumin, and creatinine; HBV DNA; and abdominal ultrasound examination. Monitoring was evaluated at 6-month intervals after the initiation of NA treatment until the end of the follow-up period (up to 3 years) or until treatment discontinuation, a gap of more than 45 days between NA treatments, loss to follow-up, death, or the study endpoint (June 30, 2022), whichever occurred first. To reflect real-world variations in visit schedules, each 6-month interval was defined with a ±2-month grace period. Patients were considered adherent to monitoring if at least 1 measurement was performed within each interval, including the grace period. Monitoring was assessed only during each patient's active observation period, up to treatment discontinuation or the last follow-up, rather than for a fixed 36-month duration.

In Part 2, we assessed the renal safety of each NA. A follow-up period of up to 3 years was initiated the day after the index date and continued until the occurrence of renal dysfunction, cessation of NA treatment, a gap exceeding 45 days between NA treatments, loss of participants to follow-up or death, or the conclusion of the study on June 30, 2022, whichever event occurred first. Renal dysfunction was defined as either (i) a validated reduction in eGFR of more than 30%, as confirmed by 2 separate observations, or (ii) a validated reduction in eGFR by more than 2 stages, as confirmed by 2 separate observations. To calculate eGFR, we used the Modification of Diet in Renal Disease (MDRD) formula (28). The MDRD equation was selected because it has been widely used in studies of CHB populations and is recommended in local clinical practice guidelines for evaluating renal function.

### Statistical analysis

To analyze the baseline characteristics, we calculated the descriptive statistics. Continuous variables were calculated as mean (SD) or median (interquartile range). We used analysis of variance to calculate *P* values and the Kruskal–Wallis test to assess the covariate balances between groups. Categorical variables were calculated as frequencies and percentages and compared using the χ^2^ test or Fisher exact test. In addition, standardized mean differences were calculated using Cohen's *d* to quantify the magnitude of imbalance between groups. The absolute values of standardized mean differences were reported, and detailed results are presented in Supplementary Table 1 (http://links.lww.com/CTG/B441).

Cumulative renal dysfunction–free survival was estimated using the Kaplan–Meier method and compared using log-rank tests. Univariate and multivariate Cox proportional hazard regression models were used to calculate the HRs and 95% CIs for the development of renal dysfunction. Drug exposure (ETV, TDF, or TAF) was forced into the model given its primary relevance to the study objective. Variables with *P* < 0.05 in the univariate analysis were subsequently entered into the multivariate model. Patients were analyzed in an as-treated approach; those who discontinued NA therapy or died during follow-up were censored at the time of the event. The proportional hazards assumption for Cox models was formally tested using Schoenfeld residuals, and no significant violations were detected. The proportions of missing values for key covariates were summarized in Supplementary Table 2 (http://links.lww.com/CTG/B441). The median imputation method was applied to handle missing data. We used the Statistical Analysis System, version 9.4 (SAS Institute, Cary, NC), for all data management and analysis. Two-sided *P* values of <0.05 were considered statistically significant.

### Sensitivity analyses

Sensitivity analyses were performed to validate the results. Specifically, (i) we excluded variables with missing values from the analysis; (ii) we used dummy variable adjustment to address missing values, aiming to explore the effects of different processing methods for missing values in the analysis; and (iii) to examine differences in the results arising from the use of various renal function calculation formulas, we calculated eGFR using both the CKD Epidemiology Collaboration (CKD-EPI) formula and the Cockcroft-Gault formula (29,30).

## RESULTS

### Study population

**Figure 1.**
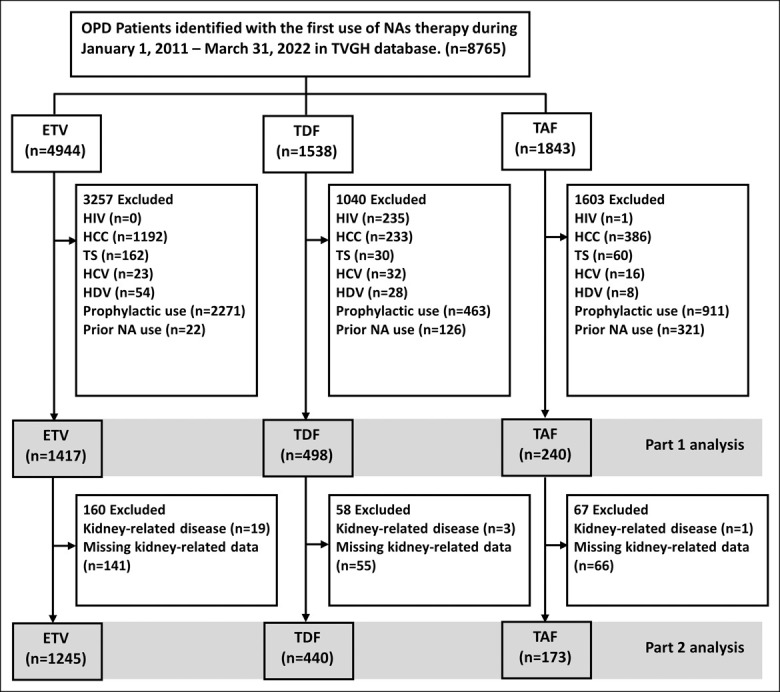
Schematic flowchart of patient selection. Numbers in boxes indicate both the number of patients excluded for each specific reason and the number of patients remaining after each selection step. ETV, entecavir; HCC, hepatocellular carcinoma; NA, nucleos(t)ide analogs; TAF, tenofovir alafenamide; TDF, tenofovir disoproxil fumarate.

Enrolment of the study population is shown in Figure 1. For Part 1, we recruited 2,155 treatment-naïve patients with CHB who received potent NAs: 1,417 (65.8%) were treated with ETV, 498 (23.1%) with TDF, and the remaining 240 (11.1%) with TAF. In Part 2, the number of participants was 1,858:1,245 who received ETV, 440 who received TDF, and 173 who received TAF.

### Baseline patient characteristics

Table 1 lists the baseline characteristics of the 3 groups included in Part 1 of the analysis. The TAF recipients (mean age, 54.6 ± 12.8 years), followed by the ETV recipients (mean age, 52.6 ± 13.1 years) and TDF recipients (mean age, 50.4 ± 13.5 years). Almost two-thirds (66.1%) of the participants were male. The mean duration of NA use was shortest for TAF (1.25 ± 0.7 years) because it was the newest drug; ETV and TDF were used, on average, 2.37 ± 0.9 years and 2.25 ± 0.9 years, respectively.

**Table 1.** Baseline demographic characteristics of the study population

	Entecavir (n = 1,417)	Tenofovir disoproxil fumarate (n = 498)	Tenofovir alafenamide (n = 240)	*P* value
Age, yr, mean (SD)	52.6 (13.1)	50.4 (13.5)	54.6 (12.8)	**<0.001**
Male, n (%)	952 (67.2)	323 (64.9)	150 (62.5)	0.290
NAs duration, yr, mean (SD)	2.37 (0.9)	2.25 (0.9)	1.25 (0.7)	**<0.001**
BMI, kg/m^2^, mean (SD)	24.9 (4.2)	24.4 (3.4)	25.8 (4.7)	**0.006**
Alcohol, n (%)	59 (12.1)	9 (7.3)	10 (13.3)	0.268
Cigarette, n (%)	147 (10.8)	47 (9.6)	30 (12.7)	0.446
Comorbidities, n (%)				
Hypertension	251 (17.7)	61 (12.3)	40 (16.7)	**0.017**
Diabetes mellitus	197 (13.9)	60 (12.1)	39 (16.3)	0.285
Dyslipidemia	191 (13.5)	70 (14.1)	31 (12.9)	0.906
Coronary arterial disease	78 (5.5)	25 (5.0)	15 (6.3)	0.786
Bone disorder	18 (1.3)	4 (0.8)	6 (2.5)	0.160
Stroke	21 (1.5)	4 (0.8)	4 (1.7)	0.475
Deyo–CCI, mean (SD)	1.8 (1.3)	1.4 (0.8)	1.6 (1.0)	**<0.001**
Liver function, median (IQR)				
ALT, U/L	102 (58–205)	109 (62–104)	108 (54–184)	0.407
AST, U/L	68 (45–121)	65.5 (39–112)	62 (39–113)	**0.032**
Albumin, g/dL	4.0 (3.3–4.4)	4.2 (3.9–4.5)	4.2 (3.8–4.5)	**<0.001**
Total bilirubin, mg/dL	0.89 (0.63–1.34)	0.79 (0.58–1.12)	0.67 (0.50–0.89)	**<0.001**
Platelet count, x10^3^/µL	173 (125–218)	193 (146–237)	178 (137–232)	**<0.001**
Prothrombin time, seconds	11.3 (10.8–12.3)	11.1 (10.7–11.6)	11.6 (11.1–12.4)	**<0.001**
FIB-4 score	2.2 (1.3–4.0)	1.8 (1.1–3.2)	2.1 (1.1–3.3)	**<0.001**
Renal function, median (IQR)				
Serum creatinine, mg/dL	0.84 (0.73–0.98)	0.81 (0.72–0.92)	0.87 (0.74–0.98)	**0.001**
BUN, mg/dL	13 (11–16)	13 (11–16)	15 (12–20)	**<0.001**
eGFR, mL/min/1.73 m^2^	90.0 (76.1–102.8)	91.7 (80.9–101.6)	85.2 (70.2–98.9)	**<0.001**
CKD stage, n (%)	n = 1,276	n = 442	n = 174	**<0.001**
Stage 1 (eGFR ≥ 90)	641 (50.2)	247 (55.9)	69 (39.7)	
Stage 2 (eGFR 60–89)	520 (40.8)	186 (42.1)	76 (43.7)	
Stage 3a (eGFR 45–59)	52 (4.1)	8 (1.8)	21 (12.1)	
Stage 3b (eGFR 30–44)	21 (1.7)	1 (0.2)	4 (2.3)	
Stage 4 (eGFR 15–29)	17 (1.3)	0 (0)	1 (0.6)	
Stage 5 (eGFR < 15)	25 (2.0)	0 (0)	3 (1.7)	
Laboratory data				
HBeAg positivity, n (%)	254 (36.3)	133 (46.8)	40 (32.0)	**0.002**
HBV DNA, log_10_ IU/mL	5.8 (4.6–7.3)	6.2 (4.4–7.6)	6.1 (4.9–7.2)	0.441
HBV DNA <2,000 IU/mL, n (%)	65 (11.1)	34 (12.5)	19 (10.6)	0.765
Alpha-fetoprotein, ng/mL	6.18 (3.8–11.7)	5.01 (3.5–9.6)	3.73 (2.2–7.6)	**0.002**
Concurrent use drugs, n (%)				
NSAIDs	115 (8.1)	30 (6.1)	18 (7.5)	0.315
Diuretics	148 (10.4)	14 (2.8)	12 (5.0)	**<0.001**

Bold entries indicate statistical significance (*P* < 0.05). ALT, alanine aminotransferase; AST, aspartate aminotransferase; BMI, body mass index; BUN, blood urea nitrogen; CKD, chronic kidney disease; Deyo–CCI, Deyo–Charlson Comorbidity Index; eGFR, estimated glomerular filtration rate; FIB-4, Fibrosis-4; HBeAg, hepatitis B e antigen; HBV, hepatitis B virus; IQR, interquartile range; NA, nucleos(t)ide analogs; NSAID, nonsteroidal anti-inflammatory drugs.

The Deyo–Charlson Comorbidity Index (Deyo–CCI) scores were higher among ETV recipients, which reflects physicians' tendency to prescribe ETV to patients with more complex comorbidities. In part 1, the eGFR was <60 mL/min/1.73 m^2^ in 115 (8.1%) ETV recipients, 9 (1.8%) TDF recipients, and 29 (12.1%) TAF recipients, indicating that physicians were less likely to prescribe TDF to patients with renal dysfunction. The baseline stage of liver fibrosis was mild to moderate, with a median Fibrosis-4 (FIB-4) score between 1.8 and 2.2.

### Monitoring behavior

The monitoring pattern after the NA treatment is illustrated in Figure 2. In Part 1, we found that ALT was monitored in more than 90% of the participants, and the percentages of the 3 groups were similar. However, serum total bilirubin levels were measured regularly in approximately 50% of patients. Albumin levels were monitored in <20% of all patients; the percentages varied by drug, and the highest proportion of patients monitored was in the ETV group. HBV DNA was monitored in approximately 50% of all patients; abdominal ultrasound studies were conducted in approximately 60%–70%; and serum creatinine levels were monitored in approximately 80% of TDF recipients, but only in 50%–70% of ETV and TAF recipients. During the first 2.5 years of follow-up, albumin monitoring rates were significantly lower in the TDF group, whereas serum creatinine monitoring rates were significantly higher compared with those in the ETV and TAF groups.

Figure 2.Monitoring rates of laboratory and imaging tests among patients receiving ETV, TDF, or TAF. Panels show the proportion of patients who underwent recommended monitoring for (**a**) alanine aminotransferase (ALT), (**b**) total bilirubin, (**c**) albumin, (**d**) hepatitis B virus DNA, (**e**) abdominal sonogram, and (**f**) serum creatinine during the 3-year follow-up period. ETV, entecavir; TAF, tenofovir alafenamide; TDF, tenofovir disoproxil fumarate.

**Figure:**
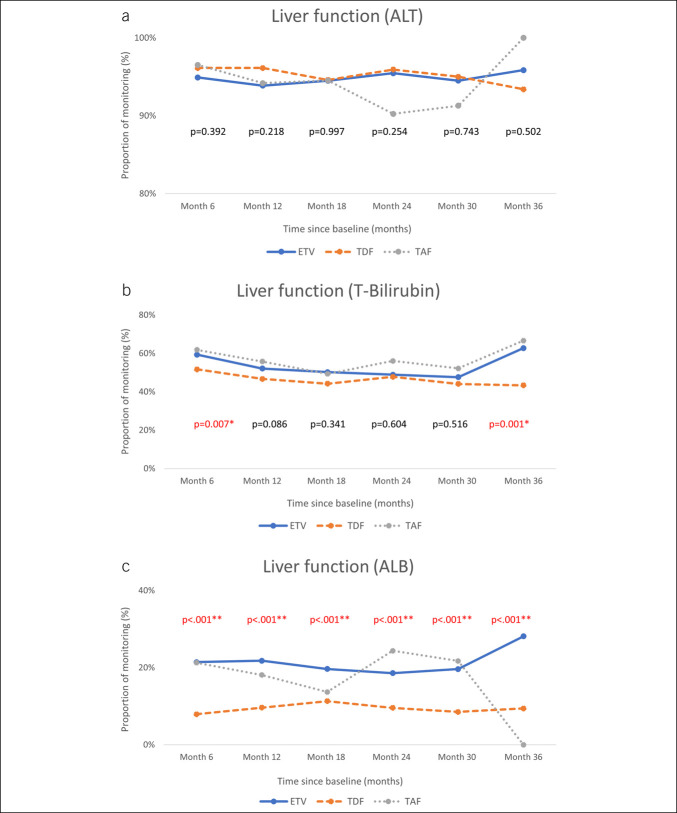

**Figure:**
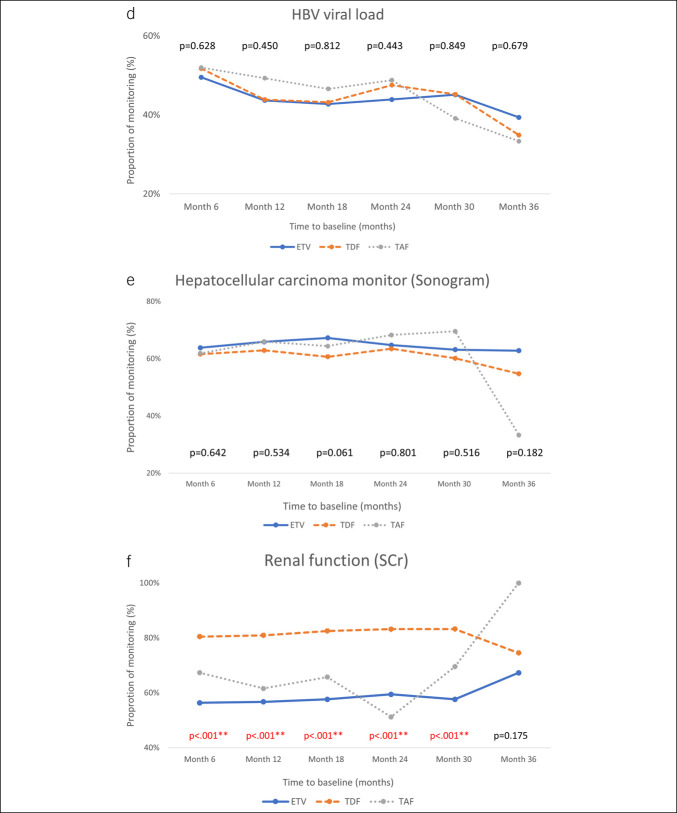

### Renal dysfunction

**Figure 3.**
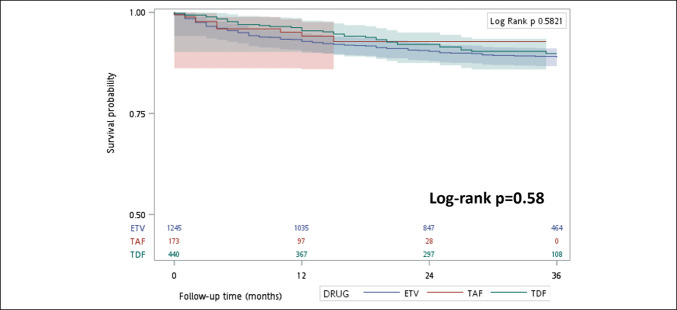
Cumulative renal dysfunction-free survival in patients with chronic hepatitis B infection receiving potent antiviral treatment. The Kaplan–Meier curves compare ETV, TDF, and TAF. Shaded areas indicate 95% confidence intervals. Numbers at risk for each group at 0, 12, 24, and 36 months are shown below the x-axis. ETV, entecavir; TAF, tenofovir alafenamide; TDF, tenofovir disoproxil fumarate.

The results of Part 2 are presented in Table 2. Renal dysfunction developed in 171 participants; the incidence per 100 person-years was 4.42 among ETV recipients, 3.85 among TDF recipients, and 4.84 among TAF recipients. As shown in Figure 3, cumulative renal dysfunction-free survival did not differ significantly among the 3 groups of patients (*P* = 0.582).

**Table 2.** Follow-up, events and incidence of renal dysfunction among patients with chronic hepatitis B who received nucleos(t)ide analogues

Drug	Follow-up duration, mo, mean (SD)	Follow-up duration, mo, median (IQR)	Events/N	Incidence rate (95% CI), events per 100 person-year
ETV	27.0 (11.7)	34.0 (17.0–36.0)	124/1,245	4.42 (3.71–5.28)
TDF	26.2 (10.9)	33.0 (19.0–35.0)	37/440	3.85 (2.79–5.32)
TAF	14.3 (8.6)	13.0 (7.0–20.0)	10/173	4.84 (2.61–9.00)

CI, confidence interval; ETV, entecavir; IQR, interquartile range; TAF, tenofovir alafenamide; TDF, tenofovir disoproxil fumarate.

The results of the univariate and multivariate analyses of the factors underlying renal dysfunction during NA therapy are listed in Table 3. The risk of renal dysfunction increased with age (HR 1.02, 95% CI 1.00–1.03, *P* = 0.008), history of stroke (HR 2.48, 95% CI 1.23–4.90, *P* = 0.009), higher Deyo–CCI scores (HR 1.22, 95% CI 1.09–1.36, *P* < 0.001), higher FIB-4 scores (HR 1.06, 95% CI 1.03–1.09, *P* < 0.001), and use of diuretics (HR 2.62, 95% CI 1.76–3.89, *P* < 0.001). Conversely, patients with higher serum albumin levels were at lower risk of renal dysfunction (HR 0.77, 95% CI 0.63–0.95, *P* = 0.015). However, NA regimens were not associated with renal dysfunction: For TDF, the adjusted HR was 1.41 (95% CI 0.95–2.08), and for TAF, the adjusted HR was 0.91 (95% CI 0.47–1.76), in comparison with ETV. Both confidence intervals were relatively wide, indicating limited statistical power, particularly for the TAF group with a smaller sample size. The comparative results for 2 of the 3 NAs are listed in Supplementary Tables 3–5 (http://links.lww.com/CTG/B441).

**Table 3.** Associated factors of renal dysfunction in 1,858 patients with chronic hepatitis B infection who received ETV, TDF, or TAF

	Univariate analysis			Multivariate analysis		
	Hazard ratio	95% CI	*P* value	Hazard ratio	95% CI	*P* value
Drug						
ETV	1	Ref		1	Ref	
TDF	0.85	(0.59, 1.23)	0.319	1.41	(0.95, 2.08)	0.088
TAF	0.80	(0.42, 1.54)	0.437	0.91	(0.47, 1.76)	0.778
Age	1.04	(1.03, 1.06)	**<0.001**	1.02	(1.00, 1.03)	**0.008**
Male	0.77	(0.57, 1.05)	0.093			
Comorbidities						
Hypertension	2.49	(1.81, 3.44)	**<0.001**	1.39	(0.96, 2.01)	0.084
Diabetes mellitus	2.10	(1.49, 2.97)	**<0.001**	0.92	(0.60, 1.40)	0.695
Dyslipidemia	1.06	(0.70, 1.59)	0.796			
CAD	1.54	(0.89, 2.67)	0.120			
Bone disorder	1.90	(0.70, 5.12)	0.205			
Stroke	4.72	(2.49, 8.93)	**<0.001**	2.48	(1.23, 4.90)	0.009
Deyo–CCI	1.52	(1.40, 1.64)	**<0.001**	1.22	(1.09, 1.36)	**<0.001**
Albumin	0.55	(0.48, 0.65)	**<0.001**	0.77	(0.63, 0.95)	**0.015**
Total bilirubin	1.07	(1.02, 1.14)	**0.014**	0.99	(0.91, 1.08)	0.803
Prothrombin time	1.19	(1.13, 1.25)	**<0.001**	1.05	(0.97, 1.13)	0.265
FIB-4 score	1.07	(1.06, 1.09)	**<0.001**	1.06	(1.03, 1.09)	**<0.001**
eGFR	1.00	(1.00, 1.01)	0.652			
NSAIDs	1.31	(0.80, 2.16)	0.292			
Diuretics	6.11	(4.41, 8.47)	**<0.001**	2.62	(1.76, 3.89)	**<0.001**

Bold entries indicate statistical significance (*P* < 0.05). CAD, coronary artery disease; CI, confidence interval; Deyo–CCI, Deyo–Charlson Comorbidity Index; eGFR, estimated glomerular filtration rate; ETV, entecavir; FIB-4, fibrosis-4; HR, hazard ratio; NSAID, nonsteroidal anti-inflammatory drugs; TAF, tenofovir alafenamide; TDF, tenofovir disoproxil fumarate.

### Sensitivity analyses

**Figure 4.**
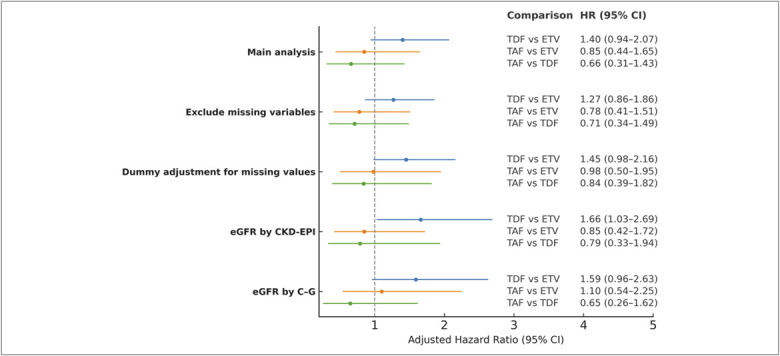
Sensitivity analyses of renal dysfunction risk by nucleos(t)ide analog comparison among patients receiving ETV, TDF, or TAF. C-G, Cockcroft-Gault; CKD, chronic kidney disease; eGFR, estimated glomerular filtration rate; ETV, entecavir; HR, hazard ratio; TAF, tenofovir alafenamide; TDF, tenofovir disoproxil fumarate.

The results of the various sensitivity analyses remained consistent; notably, the risk of renal dysfunction was significantly higher with TDF than with ETV when eGFR was calculated using the CKD-EPI formula (HR 1.66, 95% CI 1.03–2.69; Figure 4).

## DISCUSSION

In this study, we investigated the real-world clinical practice of monitoring patients who received NA therapy for CHB. ALT monitoring aligned with the recommendations, whereas HBV DNA and ultrasound surveillance did not (5,6). We observed no difference in the risk of renal dysfunction among the patients who received ETV, TDF, and TAF. Risk factors associated with renal dysfunction during NA therapy included age, history of stroke, higher Deyo–CCI scores, higher FIB-4 scores, use of diuretics, and low serum albumin levels, but not NA regimens themselves.

The European Association for the Study of the Liver recommends liver biochemistry testing every 3–4 months during the first year of NA treatment for CHB, and every 6 months thereafter (6). Moreover, serum HBV DNA levels should be tested every 3–4 months during the first year and every 6–12 months thereafter. The Asian-Pacific Association for the Study of the Liver recommends monitoring serum ALT levels every 3 months and HBV DNA levels every 3–6 months during NA therapy (5).

Regular liver ultrasound monitoring is crucial for detecting the advanced stages of liver diseases, such as liver cirrhosis or HCC, in patients with CHB (5–7,31). Regular ultrasound surveillance for patients with chronic viral hepatitis or cirrhosis improves early HCC detection, increases curative treatment rates, and enhances overall survival (32,33). The American Association for the Study of Liver Diseases suggests using ultrasonography with or without alpha-fetoprotein testing to monitor for HCC every 6 months in all patients with CHB (7).

In real-world practice, however, adherence to monitoring and surveillance guidelines for HCC is inadequate (10,11,34). A US nationwide study of 55,317 patients with CHB reported testing rates of 82% for ALT, 69% for HBV DNA, 60% for alpha-fetoprotein, and 61% for liver imaging (11). Moreover, during the follow-up period, regular serum ALT, HBV DNA, and creatinine levels were tested every 12 months in 40.3%, 28.0%, and 40.6% of the patients, respectively. Notably, even in patients at high risk for HCC, such as those with cirrhosis, male patients older than 40 years and female patients older than 50 years, fewer than 40% underwent annual monitoring for HCC.

In our cohort, more than 90% of patients underwent regular serum ALT tests after receiving NAs in accordance with the recommendations of the guidelines. However, serum HBV DNA was tested in only approximately 50% of patients. Low rates of HBV DNA monitoring might be attributable to Taiwan's national health insurance policy, which discontinues coverage for NAs once serum HBV DNA is undetectable for 1 year. Consequently, patients must either discontinue medication or bear the costs themselves. This leads clinicians to refrain from regularly monitoring HBV DNA so that patients can continue medication. Virological breakthroughs because of drug-resistant mutations are uncommon in patients receiving potent NAs for CHB (7). Hence, physicians tend to decrease the frequency of HBV DNA testing during potent NA therapy.

According to the current international management guidelines, renal function should be monitored in patients receiving NAs (5–7). In our study, the rate of renal function monitoring was higher among TDF recipients than among ETV or TAF recipients, which indicates that physicians were cognizant of possible renal adverse events of TDF. This finding is consistent with the recommendations of the Asian-Pacific Association for the Study of the Liver, which suggest monitoring renal function every 3 months if TDF is prescribed (5).

In our cohort, fewer than 70% of patients underwent semiannual HCC surveillance. These rates and those of HBV DNA testing should be improved. These findings underscore the need to bridge the gap between clinical practice and guideline recommendations. Incorporating clinical decision support tools into electronic medical records to provide clinicians with useful reminders or incentives provided by the pay-for-performance program (35,36) may improve adherence to guidelines for better management of CHB.

Several surveillance rates (e.g., liver biochemistry tests, HBV DNA tests, liver ultrasound studies, and serum creatinine measurements) were lower than those reported by Siakavellas et al. (12) These differences may be attributed to variations in the conditions of the study population and the health care system. It may be possible to reduce the frequency of monitoring patients with CHB who are at low risk for liver dysfunction (11,37,38). Our study included a relatively younger and healthier population with a lower risk of cirrhosis or HCC, and we speculate that the relevant monitoring may have been reduced as a result.

In addition to risk factors established in prior studies, such as age, comorbidities, baseline eGFR, low albumin levels, and diuretic usage (15–17,39,40), a high FIB-4 score was validated as a potential risk factor for renal dysfunction during NA therapy in this study. This finding aligns with previous studies identifying liver cirrhosis as a risk factor for renal dysfunction during TDF or ETV treatment for CHB (41). The inclusion of the FIB-4 score among the risk factors will further influence the choice of treatment and clarify the need for regular monitoring in this specific patient population.

In this study, we observed no significant difference in the risk of renal dysfunction among the ETV, TDF, and TAF recipients with CHB. These outcomes differed from those of other investigations, possibly because we excluded patients who received NAs for prophylaxis against HBV reactivation and pre-existing renal disease. As a result, our population comprised individuals with CHB who were otherwise healthy and had relatively good hepatic and renal functions.

In addition, in our study, the incidence of renal dysfunction in association with both ETV (14,42–44) and TAF (14,23,43,45) was comparable with the findings of other studies, despite variations in study designs. By contrast, the incidence of renal dysfunction during TDF treatment in our study was lower than that previously reported (14,44). The divergence in these rates may be attributable to methodological differences across studies, including variations in study duration, patient selection, baseline renal status, and definitions or estimation methods of renal function. Wong et al defined renal dysfunction as an increase in CKD stage by at least 1 stage, a ≥ 20% decrease in eGFR, or a ≥ 25% increase in serum creatinine levels and reported corresponding incidences of 15.8, 6.8 and 6.5 per 100 person-years, respectively (44). To mitigate potential overestimation due to transient fluctuations, we adopted a more conservative definition of renal dysfunction According to the Kidney Disease Improving Global Outcomes 2024 guideline, eGFR changes greater than 20% exceed expected biological variation and declines of 30%–40% are considered clinically meaningful surrogates for kidney failure risk, supporting our threshold choice (46). This stricter definition may have led to a lower observed incidence. Given the variability in definitions across studies, a standardized and clinically meaningful criterion for assessing renal function change during long-term antiviral therapy warrants further validation in future research.

The method used to calculate renal function can significantly affect the interpretation of study outcomes. When using the MDRD formula or the Cockcroft-Gault formula to evaluate renal function, we found no notable difference among the 3 potent NAs; however, when using the CKD-EPI formula for the same purpose, we observed that the risk of renal dysfunction was slightly higher with TDF than with ETV. This variation in outcomes highlights how results can reflect the choice of calculation method, as in previous studies (47,48). Different formulas may result in differing perceptions of the renal effects of NAs. Therefore, the findings of this study should be interpreted with caution. Further investigation of the mechanisms contributing to these differing results could lead to valuable insights for clinical practice and future research.

Our study has several strengths. First, we conducted head-to-head analyses to compare the effects of 3 first-line NAs. This approach identified associated factors and enabled more definitive conclusions regarding drug effectiveness and safety, thereby helping to optimize therapy. Second, the results from real-world settings are highly generalizable, indicating the need for surveillance and renal safety of NAs. Third, detailed laboratory (e.g., serum creatinine level and eGFR) and demographic data improved the accuracy of our results. Fourth, we explored the effects of different scenarios in the sensitivity analyses to assess the robustness of the results.

Despite these strengths, this study has several limitations. First, in this retrospective observational study, we could not account for all residual or unmeasured confounders, such as adherence to medication. Channeling bias was also inevitable, as physicians' prescribing decisions were influenced by patient age, comorbidities, and baseline renal function. Although we adjusted for these factors in multivariable models and performed sensitivity analyses, residual confounding cannot be entirely excluded. In addition, missing laboratory data may have introduced potential bias. Although median imputation and sensitivity analyses were conducted to mitigate this issue, missingness remains an inherent limitation of real-world retrospective datasets. Second, our data were collected from a single center, which might have led to both underestimations and introduced a selection bias, thereby limiting the generalizability of our findings. Differences in institutional practice patterns, patient characteristics, and laboratory monitoring policies across hospitals may further restrict the applicability of our results to other settings. Third, because TAF was the newest NA available, the follow-up period for TAF was shorter and the sample size was smaller than those for ETV and TDF. Consequently, the adjusted HRs for TDF and TAF vs ETV were not statistically significant, and the wide confidence intervals indicate limited statistical power for detecting differences in renal outcomes. This limited observation period and smaller TAF cohort may have reduced the ability to detect rare adverse events or long-term renal outcomes. Future multicenter studies with larger sample size, longer follow-up durations, and cost-effectiveness analyses of improved renal monitoring are warranted to validate and extend our findings.

In this real-world cohort, adherence to recommended monitoring for patients with CHB on NAs was suboptimal. ETV, TDF, and TAF demonstrated comparable renal safety profiles over 3 years. Advancing age, comorbidities, stage of liver fibrosis, use of diuretics, and low serum albumin levels, but not NA regimens, are risk factors associated with renal dysfunction during NA therapy.

## CONFLICTS OF INTEREST

**Guarantor of the article:** Chien-Wei Su, MD, PhD.

**Specific author contributions:** C.-C.H.: Conceptualization, methodology, data curation, investigation, writing—original draft, writing—review and editing, and funding acquisition. Z.-E.C.: Conceptualization, methodology, formal analysis, investigation, visualization, writing—original draft. F.-H.C.: Conceptualization, methodology, investigation, writing—review, and editing. C.-Y.C.: Methodology, resources, and supervision. Y.-L.C.: Methodology, resources, and supervision. Y-CC.: Project Administration, Validation, and Supervision. M.-C.H.: Project administration, validation, and supervision. J.-C.W.: Project administration, validation, and supervision. C.-W.S.: Conceptualization, methodology, investigation, validation, supervision writing—review and editing, and funding acquisition. C.-W.S. had full access to all the data in the study and took responsibility for the integrity of the data and the accuracy of the data analysis. All authors approved the final version of the article, including the authorship list.

**Financial support:** Taipei Veterans General Hospital, Grant/Award Number: V111B-043, V111EA-017, V113C-141, Center of Excellence for Cancer Research MOHW112-TDU-B-221-124007, Y.L. Lin Hung Tai Education Foundation, and Big Data Center. National Science and Technology Council: Grant/Award Number: MOST 111-2635-B-075-001, NSTC 112-2314-B-075-043-MY2. Gilead Sciences: Grant/Award Number CO-TW-988-6355. The funders had no role in the study design; collection, analyses, or interpretation of data; writing of the manuscript; or decision to publish the results.

**Potential competing interests:** There are no potential conflicts of financial or non-financial interest in the study. Chien-Wei Su: Speaker bureau: Gilead Sciences, Bristol-Myers Squibb, AbbVie, Bayer, and Roche. Advisory arrangements: Gilead Sciences. Grants: Bristol-Myers Squibb, Gilead Sciences, and Eiger. Part of this study was presented as a poster exhibition at the annual meeting of the Asian Pacific Association for the Study of the Liver (APASL), Kyoto, Japan, on March 27-31, 2024.

**Data availability statement:** The data generated and examined as part of this study cannot be made publicly accessible because of the regulations set by the Institutional Review Board of Taipei Veterans General Hospital. However, researchers can request access to the dataset by contacting the corresponding author or the Institutional Review Board of Taipei Veterans General Hospital (email: irbopinion@vghtpe.gov.tw). The use of these data is restricted to research purposes.

**Study Highlights**

**WHAT IS KNOWN**

✓ Entecavir, tenofovir disoproxil fumarate, and tenofovir alafenamide are first-line nucleos(t)ide analogs (NAs) for chronic hepatitis B.
✓ Information about monitoring practices for NAs is limited.
✓ The effects of NAs on renal safety have remained inconclusive.

**WHAT IS NEW HERE**

✓ Real-world alanine aminotransferase monitoring ≥90%, but other recommended tests were not performed regularly.
✓ The risks of renal dysfunction were similar for entecavir, tenofovir disoproxil fumarate, and tenofovir alafenamide.

## Supplementary Material

## ABBREVIATIONS:

ALT: alanine aminotransferase
AST: aspartate aminotransferase
BMI: body mass index
BUN: blood urea nitrogen
CAD: coronary artery disease
CHB: chronic hepatitis B
CI: confidence interval
CKD: chronic kidney disease
CKD-EPI: Chronic Kidney Disease Epidemiology Collaboration
Deyo–CCI: Deyo–Charlson Comorbidity Index
eGFR: estimated glomerular filtration rate
ETV: entecavir
FIB-4: fibrosis-4
HBV: hepatitis B virus
HBeAg: hepatitis B e antigen
HCC: hepatocellular carcinoma
HDV: hepatitis D virus
HR: hazard ratio
IQR: interquartile range
KDIGO: Kidney Disease Improving Global Outcomes
MDRD: Modification of Diet in Renal Disease
NA: nucleos(t)ide analog
NSAID: nonsteroidal anti-inflammatory drug
OPD: outpatient department
TAF: tenofovir alafenamide
TDF: tenofovir disoproxil fumarate
