# Supplementary material for: Monitoring Adherence and Renal Safety of Nucleos(t)ide Analogs for Patients With Chronic Hepatitis B
Source: Clin Transl Gastroenterol. 2025 Dec 9;17(2):e00962. doi: 10.14309/ctg.0000000000000962 (PMC12922928; doi:10.14309/ctg.0000000000000962)
Supplement: Supplementary file 1 [file ct9-17-e00962-s001.docx]

**Supplementary TABLE 1** **Baseline demographic characteristics of the study population with standardized mean differences (SMDs)**

|  | ETV  (n=1,417) | TDF  (n=498) | TAF  (n=240) | *P* value | SMD | | |
| --- | --- | --- | --- | --- | --- | --- | --- |
|  |  |  |  |  | ETV vs TDF | ETV vs TAF | TFD vs TAF |
| Age, yr, mean (SD) | 52.6 (13.1) | 50.4 (13.5) | 54.6 (12.8) | **<.001** | 0.17 | 0.15 | **0.31** |
| Male, n (%) | 952 (67.2) | 323 (64.9) | 150 (62.5) | 0.290 | 0.05 | 0.04 | 0.01 |
| NAs duration, yr, mean (SD) | 2.37 (0.9) | 2.25 (0.9) | 1.25 (0.7) | **<.001** | 0.09 | **0.72** | **0.79** |
| BMI, kg/m^2^, mean (SD) | 24.9 (4.2) | 24.4 (3.4) | 25.8 (4.7) | **0.006** | 0.17 | **0.22** | 0.05 |
| Alcohol, n (%) | 59 (12.1) | 9 (7.3) | 10 (13.3) | 0.268 | 0.14 | 0.19 | 0.05 |
| Cigarette, n (%) | 147 (10.8) | 47 (9.6) | 30 (12.7) | 0.446 | 0.14 | 0.05 | 0.09 |
| Comorbidities, n (%) |  |  |  |  |  |  |  |
| Hypertension | 251 (17.7) | 61 (12.3) | 40 (16.7) | **0.017** | 0.14 | 0.03 | 0.12 |
| Diabetes mellitus | 197 (13.9) | 60 (12.1) | 39 (16.3) | 0.285 | 0.08 | 0.03 | 0.05 |
| Dyslipidemia | 191 (13.5) | 70 (14.1) | 31 (12.9) | 0.906 | 0.10 | 0.08 | 0.03 |
| Coronary arterial disease | 78 (5.5) | 25 (5.0) | 15 (6.3) | 0.786 | 0.08 | 0.05 | 0.03 |
| Bone disorder | 18 (1.3) | 4 (0.8) | 6 (2.5) | 0.160 | 0.08 | 0.06 | 0.02 |
| Stroke | 21 (1.5) | 4 (0.8) | 4 (1.7) | 0.475 | 0.06 | 0.05 | 0.01 |
| Deyo’s CCI, mean (SD) | 1.8 (1.3) | 1.4 (0.8) | 1.6 (1.0) | **<.001** | 0.07 | 0.08 | 0.01 |
| Liver function, median (IQR) |  |  |  |  |  |  |  |
| ALT, U/L | 102 (58-205) | 109 (62-104) | 108 (54-184) | 0.407 | 0.01 | 0.11 | 0.12 |
| AST, U/L | 68 (45-121) | 65.5 (39-112) | 62 (39-113) | **0.032** | 0.07 | 0.15 | 0.10 |
| Albumin, g/dL | 4.0 (3.3-4.4) | 4.2 (3.9-4.5) | 4.2 (3.8-4.5) | **<.001** | **0.27** | **0.23** | 0.01 |
| Total bilirubin, mg/dL | 0.89 (0.63-1.34) | 0.79 (0.58-1.12) | 0.67 (0.50-0.89) | **<.001** | **0.23** | **0.35** | **0.28** |
| Platelet count, 1000/$\mu$L | 173 (125-218) | 193 (146-237) | 178 (137-232) | **<.001** | **0.31** | 0.05 | **0.45** |
| Prothrombin time, second | 11.3 (10.8-12.3) | 11.1 (10.7-11.6) | 11.6 (11.1-12.4) | **<.001** | **0.31** | 0.15 | 0.06 |
| FIB-4 score | 2.2 (1.3-4.0) | 1.8 (1.1-3.2) | 2.1 (1.1-3.3) | **<.001** | **0.20** | 0.00 | 0.15 |
| Renal function, median (IQR) |  |  |  |  |  |  |  |
| Serum creatinine, mg/dL | 0.84 (0.73-0.98) | 0.81 (0.72-0.92) | 0.87 (0.74-0.98) | **0.001** | **0.20** | 0.17 | **0.22** |
| BUN, mg/dL | 13 (11-16) | 13 (11-16) | 15 (12-20) | **<.001** | **0.22** | **0.21** | **0.66** |
| eGFR, mL/min/1.73 m^2^ | 90.0 (76.1-102.8) | 91.7 (80.9-101.6) | 85.2 (70.2-98.9) | **<.001** | 0.16 | 0.16 | **0.43** |
| CKD stage, n (%) | n=1276 | n=442 | n=174 | **<.001** | **0.35** | **0.38** | **0.59** |
| Stage 1 (eGFR ≥ 90) | 641 (50.2) | 247 (55.9) | 69 (39.7) |  |  |  |  |
| Stage 2 (eGFR 60-89) | 520 (40.8) | 186 (42.1) | 76 (43.7) |  |  |  |  |
| Stage 3a (eGFR 45-59) | 52 (4.1) | 8 (1.8) | 21 (12.1) |  |  |  |  |
| Stage 3b (eGFR 30-44) | 21 (1.7) | 1 (0.2) | 4 (2.3) |  |  |  |  |
| Stage 4 (eGFR 15-29) | 17 (1.3) | 0 (0) | 1 (0.6) |  |  |  |  |
| Stage 5 (eGFR < 15) | 25 (2.0) | 0 (0) | 3 (1.7) |  |  |  |  |
| eGFR < 60 | 115 (9.0) | 9 (2.0) | 29 (16.7) |  | **0.31** | **0.23** | **0.52** |
| Laboratory data |  |  |  |  |  |  |  |
| HBeAg positivity, n (%) | 254 (36.3) | 133 (46.8) | 40 (32.0) | **0.002** | **0.21** | 0.11 | 0.10 |
| HBV DNA, log_10_ IU/mL | 5.8 (4.6-7.3) | 6.2 (4.4-7.6) | 6.1 (4.9-7.2) | 0.441 | 0.01 | 0.13 | 0.13 |
| HBV DNA <2000 IU/mL, n (%) | 65 (11.1) | 34 (12.5) | 19 (10.6) | 0.765 | 0.06 | 0.07 | 0.01 |
| Alpha-fetoprotein, ng/mL | 6.18 (3.8-11.7) | 5.01 (3.5-9.6) | 3.73 (2.2-7.6) | **0.002** | 0.10 | 0.14 | 0.08 |
| Concurrent use drugs, n (%) |  |  |  |  |  |  |  |
| NSAIDs | 115 (8.1) | 30 (6.1) | 18 (7.5) | 0.315 | 0.08 | 0.13 | 0.05 |
| Diuretics | 148 (10.4) | 14 (2.8) | 12 (5.0) | **<.001** | **0.24** | 0.15 | 0.09 |

Abbreviations: ALT, alanine aminotransferase; AST, aspartate aminotransferase; BMI, body mass index; BUN, blood urea nitrogen; CCI, Charlson Comorbidity Index; CKD, chronic kidney disease; eGFR, estimated glomerular filtration rate; ETV, entecavir; FIB-4, Fibrosis-4 index panel; HBeAg, hepatitis B e antigen; HBV, hepatitis B virus; IQR, interquartile range; NA, nucleos(t)ide analogs; NSAID, nonsteroidal anti-inflammatory drugs; SD, standard deviation; SMD, standardized mean differences; TAF, tenofovir alafenamide; TDF, tenofovir disoproxil fumarate

**Supplementary TABLE 2** **Missing data for baseline variables**

|  | Entecavir  (n=1,417) | Tenofovir disoproxil fumarate  (n=498) | Tenofovir alafenamide  (n=240) |
| --- | --- | --- | --- |
| Age | 0 (0%) | 0 (0%) | 0 (0%) |
| Male | 0 (0%) | 0 (0%) | 0 (0%) |
| NAs duration | 0 (0%) | 0 (0%) | 0 (0%) |
| BMI | 437 (30.8%) | 159 (31.9) | 44 (18.3) |
| Alcohol | 930 (65.6%) | 374 (75.1%) | 165 (68.8%) |
| Cigarette | 59 (4.2%) | 9 (1.8%) | 4 (1.7%) |
| Liver function |  |  |  |
| ALT | 75 (5.3%) | 15 (3.0%) | 50 (20.8%) |
| AST | 86 (6.1%) | 20 (4.0%) | 50 (20.8%) |
| Albumin | 631 (44.5%) | 303 (60.8%) | 109 (45.4%) |
| Total bilirubin | 219 (15.5%) | 73 (14.7%) | 41 (17.1%) |
| Platelet count | 259 (18.3%) | 112 (22.5%) | 130 (54.2%) |
| Prothrombin time | 432 (30.5%) | 167 (33.5%) | 149 (62.1%) |
| FIB-4 score | 278 (19.6%) | 120 (24.1%) | 132 (55.0%) |
| Renal function |  |  |  |
| Serum creatinine | 141 (10.0%) | 55 (11.0%) | 66 (27.5%) |
| BUN | 460 (32.5%) | 146 (29.3%) | 135 (56.3%) |
| Laboratory data |  |  |  |
| HBeAg positivity, n (%) | 720 (50.8%) | 214 (43.0%) | 115 (47.9%) |
| HBV DNA, log_10_ IU/mL | 831 (58.6%) | 227 (45.6%) | 60 (25.0%) |
| Alpha-fetoprotein, ng/mL | 1096 (77.3%) | 403 (80.9%) | 214 (89.2%) |

Abbreviations: ALT, alanine aminotransferase; AST, aspartate aminotransferase; BMI, body mass index; BUN, blood urea nitrogen; FIB-4, Fibrosis-4; HBeAg, hepatitis B e antigen; HBV, hepatitis B virus; NA, nucleos(t)ide analogs

**Supplementary Table 3** **Factors associated with renal dysfunction in patients with chronic hepatitis B infection who received ETV or TDF**

|  | Univariate analysis | | |  | Multivariate analysis | | |
| --- | --- | --- | --- | --- | --- | --- | --- |
|  | Hazard ratio | 95% CI | P value |  | Hazard ratio | 95% CI | P value |
| Drug |  |  |  |  |  |  |  |
| ETV | 1 | (ref) |  |  | 1 | (ref) |  |
| TDF | 0.85 | 0.59-1.23 | 0.38 |  | 1.40 | 0.94-2.07 | 0.09 |
| Age | 1.04 | 1.03-1.06 | **<.0001** |  | 1.02 | 1.01-1.03 | **0.03** |
| Male | 0.76 | 0.55-1.04 | 0.08 |  |  |  |  |
| Comorbidities |  |  |  |  |  |  |  |
| Hypertension | 2.68 | 1.93-3.72 | **<.0001** |  | 1.45 | 0.98-2.14 | 0.06 |
| Diabetes mellitus | 2.23 | 1.56-3.17 | **<.0001** |  | 0.95 | 0.62-1.47 | 0.82 |
| Dyslipidemia | 1.08 | 0.71-1.65 | 0.71 |  |  |  |  |
| CAD | 1.72 | 0.99-2.97 | 0.05 |  |  |  |  |
| Bone disorder | 2.35 | 0.87-6.34 | 0.09 |  |  |  |  |
| Stroke | 4.62 | 2.36-9.05 | **<.0001** |  | 2.36 | 1.15-4.81 | **0.02** |
| Deyo–CCI | 1.52 | 1.41-1.65 | **<.0001** |  | 1.23 | 1.10-1.37 | **0.0003** |
| Albumin | 0.55 | 0.47-0.65 | **<.0001** |  | 0.79 | 0.63-0.99 | **0.04** |
| Total bilirubin | 1.07 | 1.01-1.14 | **0.02** |  | 0.99 | 0.91-1.08 | 0.93 |
| Prothrombin time | 1.18 | 1.18-1.24 | **<.0001** |  | 1.03 | 0.95-1.13 | 0.44 |
| FIB-4, score | 1.12 | 1.09-1.15 | **<.0001** |  | 1.05 | 1.01-1.10 | **0.027** |
| eGFR | 1.00 | 0.99-1.01 | 0.36 |  |  |  |  |
| NSAIDs | 1.33 | 0.80-2.23 | 0.28 |  |  |  |  |
| Diuretics | 6.28 | 4.49-8.78 | **<.0001** |  | 2.76 | 1.83-4.17 | **<.0001** |

Abbreviations: CAD, Coronary artery disease; Deyo–CCI, Deyo–Charlson Comorbidity Index; CI, confidence interval; eGFR, estimated glomerular filtration rate; ETV, entecavir; FIB-4, Fibrosis-4; HR, hazard ratio; NSAID, nonsteroidal anti-inflammatory drug; TDF, tenofovir disoproxil fumarate

**Supplementary Table 4** **Factors associated with renal dysfunction in patients with chronic hepatitis B infection who received ETV or TAF**

|  | Univariate analysis | | |  | Multivariate analysis | | |
| --- | --- | --- | --- | --- | --- | --- | --- |
|  | Hazard ratio | 95% CI | P value |  | Hazard ratio | 95% CI | P value |
| Drug |  |  |  |  |  |  |  |
| ETV | ref |  |  |  | ref |  |  |
| TAF | 0.77 | 0.40-1.47 | 0.43 |  | 0.85 | 0.44-1.65 | 0.62 |
| Age | 1.05 | 1.03-1.06 | **<.001** |  | 1.02 | 1.01-1.04 | **0.003** |
| Male | 0.77 | 0.54-1.09 | 0.14 |  |  |  |  |
| Comorbidities |  |  |  |  |  |  |  |
| Hypertension | 2.25 | 1.57-3.24 | **<.001** |  | 1.17 | 0.77-1.77 | 0.46 |
| Diabetes mellitus | 2.17 | 1.47-3.19 | **<.001** |  | 0.97 | 0.60-1.55 | 0.89 |
| Dyslipidemia | 0.77 | 0.46-1.30 | 0.33 |  |  |  |  |
| CAD | 1.91 | 1.10-3.32 | **0.02** |  | 1.11 | 0.61-2.01 | 0.73 |
| Bone disorder | 1.03 | 0.26-4.16 | 0.97 |  |  |  |  |
| Stroke | 4.42 | 2.21-9.24 | **<.001** |  | 2.72 | 1.24-5.97 | **0.01** |
| Deyo–CCI | 1.50 | 1.37-1.63 | **<.001** |  | 1.19 | 1.06-1.34 | **0.003** |
| Albumin | 0.54 | 0.46-0.64 | **<.001** |  | 0.74 | 0.60-0.92 | **0.006** |
| Total bilirubin | 1.08 | 1.02-1.14 | **0.006** |  | 1.00 | 0.92-1.09 | 0.92 |
| Prothrombin time | 1.19 | 1.13-1.25 | **<.001** |  | 1.06 | 0.98-1.15 | 0.14 |
| FIB-4, score | 1.07 | 1.06-1.09 | **<.001** |  | 1.05 | 1.02-1.08 | **<.001** |
| eGFR | 0.997 | 0.990-1.004 | 0.442 |  |  |  |  |
| NSAIDs | 1.37 | 0.80-2.35 | 0.245 |  |  |  |  |
| Diuretics | 6.07 | 4.25-8.66 | **<.001** |  | 2.35 | 1.53-3.59 | **<.001** |

Abbreviations: CAD, Coronary artery disease; Deyo–CCI, Deyo–Charlson Comorbidity Index; CI, confidence interval; eGFR, estimated glomerular filtration rate; ETV, entecavir; FIB-4, Fibrosis-4; HR, hazard ratio; NSAID, nonsteroidal anti-inflammatory drug; TAF, Tenofovir alafenamide

**Supplementary Table 5** **Factors associated with renal dysfunction in patients with chronic hepatitis B infection who received TDF or TAF**

|  | Univariate analysis | | |  | Multivariate analysis | | |
| --- | --- | --- | --- | --- | --- | --- | --- |
|  | Hazard ratio | 95% CI | P value |  | Hazard ratio | 95% CI | P value |
| Drug |  |  |  |  |  |  |  |
| TDF | ref |  |  |  | ref |  |  |
| TAF | 1.07 | 0.52-2.18 | 0.86 |  | 0.66 | 0.31-1.43 | 0.29 |
| Age | 1.04 | 1.02-1.06 | **<.001** |  | 1.01 | 0.99-1.04 | 0.36 |
| Male | 0.80 | 0.45-1.43 | 0.45 |  |  |  |  |
| Comorbidities |  |  |  |  |  |  |  |
| Hypertension | 2.66 | 1.42-4.98 | **0.002** |  | 1.88 | 0.91-3.88 | 0.09 |
| Diabetes mellitus | 1.60 | 0.79-3.21 | 0.19 |  |  |  |  |
| Dyslipidemia | 1.92 | 0.99-3.71 | 0.05 |  |  |  |  |
| CAD | 0.00 | 0.00-0.00 | 0.98 |  |  |  |  |
| Bone disorder | 3.66 | 0.88-15.16 | 0.07 |  |  |  |  |
| Stroke | 5.66 | 1.76-18.25 | **<.001** |  | 2.36 | 0.63-8.78 | 0.20 |
| Deyo–CCI | 1.65 | 1.31-2.07 | **<.001** |  | 1.29 | 0.97-1.73 | 0.07 |
| Albumin | 0.63 | 0.43-0.94 | **0.02** |  | 0.89 | 0.58-1.38 | 0.61 |
| Total bilirubin | 0.78 | 0.44-1.39 | 0.40 |  |  |  |  |
| Prothrombin time | 1.22 | 1.03-1.44 | **0.02** |  | 1.06 | 0.85-1.32 | 0.59 |
| FIB-4, score | 1.07 | 1.04-1.10 | **<.001** |  | 1.07 | 1.04-1.11 | **<.001** |
| eGFR | 1.01 | 0.99-1.03 | 0.09 |  |  |  |  |
| NSAIDs | 0.99 | 0.31-3.18 | 0.98 |  |  |  |  |
| Diuretics | 5.58 | 2.49-12.49 | **<.001** |  | 3.15 | 1.20-8.30 | **0.02** |

Abbreviations: CAD, Coronary artery disease; Deyo–CCI, Deyo–Charlson Comorbidity Index; CI, confidence interval; eGFR, estimated glomerular filtration rate; FIB-4, Fibrosis-4; HR, hazard ratio; NSAID, nonsteroidal anti-inflammatory drug; TAF, Tenofovir alafenamide; TDF, tenofovir disoproxil fumarate
